# Treatment of Diabetic Mice with a Combination of Ketogenic Diet and Aerobic Exercise via Modulations of PPARs Gene Programs

**DOI:** 10.1155/2018/4827643

**Published:** 2018-03-20

**Authors:** Qiang Zhang, Lingyan Xu, Jie Xia, Dongmei Wang, Min Qian, Shuzhe Ding

**Affiliations:** ^1^Key Laboratory of Adolescent Health Assessment and Exercise Intervention, Ministry of Education, East China Normal University, Shanghai 200241, China; ^2^School of Physical Education & Health Care, East China Normal University, Shanghai 200241, China; ^3^Shanghai Key Laboratory of Regulatory Biology, Institute of Biomedical Sciences and School of Life Sciences, East China Normal University, Shanghai, China

## Abstract

Type 2 diabetes is a prevalent chronic disease arising as a serious public health problem worldwide. Diet intervention is considered to be a critical strategy in glycemic control of diabetic patients. Recently, the low-carbohydrate ketogenic diet is shown to be effective in glycemic control and weight loss. However, hepatic lipid accumulation could be observed in mice treated with ketogenic diet. On the other hand, exercise is a well-known approach for treating nonalcoholic fatty liver disease. We thus hypothesize that the combination of ketogenic diet and exercise could improve insulin sensitivity, while minimizing adverse effect of hepatic steatosis. In order to test this hypothesis, we established diabetic mice model with streptozotocin (STZ) and divided them into control group, ketogenic diet group, and ketogenic diet with aerobic exercise group. We found that after six weeks of intervention, mice treated with ketogenic diet and ketogenic diet combined with exercise both have lower body weights, HbAlc level, HOMA index, and improvements in insulin sensitivity, compared with diabetes group. In addition, mice in ketogenic diet intervention exhibited hepatic steatosis shown by serum and hepatic parameters, as well as histochemistry staining in the liver, which could be largely relieved by exercise. Furthermore, gene analysis revealed that ketogenic diet in combination with exercise reduced PPAR*γ* and lipid synthetic genes, as well as enhancing PPAR*α* and lipid *β*-oxidation gene program in the liver compared to those in ketogenic diet without exercise. Overall, the present study demonstrated that the combination of ketogenic diet and a moderate-intensity aerobic exercise intervention improved insulin sensitivity in diabetic mice, while avoiding hepatic steatosis, which provided a novel strategy in the combat of diabetes.

## 1. Introduction

Type 2 diabetes (T2D) has become a worldwide epidemic that affected over 422 million people in 2014 according to the World Health Organization. Uncontrolled increased blood glucose concentration is a hallmark of T2D that can lead to long-term complications including micro- and macrocardiovascular complications, neuropathy, kidney failure, and increased risk of cancer development [1–4]. Maintaining blood glucose within a normal range is the goal in the treatment of T2D, as this dramatically reduces the risk of diabetes-associated complications [5–9]. Fasting hyperglycemia, due to the increased rates of hepatic gluconeogenesis, and the insulin resistance are the hallmark of T2D, while the current treatment is ineffective [3, 10, 11].

Although antidiabetic drugs, such as thiazolidinedione and metformin, have been widely used in treating T2D, a growing number of nondrug therapies have spread with considerable attention to drug safety [12]. Among these strategies, nutritional therapy has been recommended by the American Diabetes Association for all people with diabetes [13]. In particular, a very low-carbohydrate/high-fat diet (ketogenic diet, KD) is one of the most used dietary therapies for patients with diabetes or obesity in recent clinical studies [14–16]. The classic ketogenic diet (KD) is a high-fat, low-carbohydrate diet that induces ketone body production through fat metabolism with the goal of mimicking a fasting state in the metabolic tissues and shifting the predominant caloric source from carbohydrate to fat [17]. Indeed, previous studies reported that the ketogenic diet contributed to weight loss in diet-induced obese patients as well as improving glycemic control and metabolic parameters in type 2 diabetes [18–22].

However, the potential side effects of ketogenic diet of inducing dyslipidemia as well as hepatic steatosis and fibrosis by driving alterations in hepatic glucose levels, lipid metabolism, and inflammation status limit its applications [23–25]. On the other hand, most researches thought exercise could prevent hepatic steatosis, while the molecular mechanism for this alteration is unknown [26–29]. Thus, we hypothesize that the combination of ketogenic diet and exercise may improve insulin sensitivity, while minimizing the adverse effect of hepatic lipid disorders. In order to test this hypothesis, we established diabetic mice model with streptozotocin (STZ) and divided them into control group (diabetes control, high-fat diet), ketogenic diet group (diabetes + ketogenic diet), and ketogenic diet with aerobic exercise group (diabetes + ketogenic diet + aerobic exercise training), based on different diet and interventions. After 6-week intervention, we found that diabetic mice in ketogenic diet intervention significantly improved insulin sensitivity while exhibiting hepatic steatosis, which could be largely relieved by aerobic exercise. In addition, gene analysis revealed that modulations of PPAR*α* and PPAR*γ* gene programs in the liver were potentially responsible for the effects of the combination intervention. Overall, our results suggested that a combination of ketogenic diets and aerobic exercise could be a better strategy targeting diabetes.

## 2. Materials and Methods

### 2.1. Animals

All animal procedures were approved by the Experimental Animal Care and Use Committee of East China Normal University (M20170316, Shanghai, China) and conducted in accordance with the Guide for the Care and Use of Laboratory Animals (NIH publication, 8th edition, 2011). Three-week-old male C57BL/6J mice were obtained from Shanghai Laboratory Animal Company (SLAC, Shanghai, China). Mice were maintained in a specific pathogen-free animal facility with a temperature-regulated fashion (22°C ± 2°C) on a 12 h light/12 h dark cycle. Mice had free access to pure water and fed with chow diet, high-fat diet, or ketogenic diet. Body weight and the water and food intake were measured once a week throughout the intervention until the end of experiment [30].

### 2.2. Type 2 Diabetic Mouse Model and Interventions

Three-week-old male C57BL/6J mice were used for inducing type 2 diabetes. Mice were fed a high-fat diet (SLAC) for 4 weeks and then were given streptozotocin (STZ, Sigma-Aldrich, St. Louis, MO, USA) by intraperitoneal injection at a dose of 100 mg/kg in freshly prepared sodium citrate buffer (pH 4.4–4.5) [31]. An injection of sodium citrate buffer alone served as a control. The fast blood glucose level of mice was measured once a week using the blood from the tail vein and glucose monitor (ACCU-CHEK Active, Roche Basel, Switzerland). After STZ injection, mice with a fasting blood glucose level higher than 11.1 mmol/L on 3 consecutive weeks were considered as type 2 diabetic mice and divided into 3 subgroups, diabetic group (diabetes control) receiving high-fat diet, diabetes + KD group, and diabetes + KD + AE (aerobic exercise training) group receiving a ketogenic diet. The ingredient composition of all diet fed to mice was listed in Supplementary Table 1.

### 2.3. Exercise Performance Test and Exercise Training Protocol

The mice from diabetes + KD + AE group were placed on a motor-driven rodent treadmill (ZH-PT, Hangzhou, China). After an adaptation of treadmill, mice were subjected to an exercise performance test. First, mice warmed up at a speed of 8 m/min for 5 minutes, and then speed was increased by steps of 2 m/min every 2 min until the mice were unable or unwilling to carry on despite mild stimulation with a wooden cane. Performance tests were carried out at the beginning and 4 weeks later of training in order to readjust training intensity.

Aerobic exercise protocols were adapted from previous works described by Hafstad et al. [32], with the following modifications. For exercise acclimatization, all mice were conditioned at a speed of 8 m/min, 10 min/day, for 5 days to be familiar with the treadmill environment. After this period, mice ran for 60 min at intensity of 50–60% (0% slope) with the maximum speed reached during the last exercise performance test. Exercise animal group were exposed to 8 weeks of training consisting of treadmill running 5 days per week, while the sedentary animal group stayed in their home cage throughout the course of the experiment. The room temperature was maintained at 22 ± 2°C during the training sessions. After 8 weeks of training, animals were fasted overnight and sacrificed before tissues were harvested.

### 2.4. Glucose Tolerance Test and Insulin Tolerance Test

GTT (intraperitoneal glucose tolerance test) and ITT (intraperitoneal Insulin tolerance test) were performed in vivo at the end of the 6-week exercise training period. After overnight fasting or 4 hours, venous blood was collected from the tail for measurement of baseline glucose level at* t* = 0 min, and then at* t* = 15, 30, 60, 90, 120, and 180 min after an intraperitoneal injection of glucose (Sigma-Aldrich, St. Louis, MO, USA) 1 g/kg body weight or insulin (Novolin R; Novo Nordisk) 0.75 U/kg body weight, blood glucose was measured using ACCU-CHEK Active.

### 2.5. Blood and Serum Assays

Whole blood was collected in EDTA coated tubes for HbA1c analysis with a glycosylated hemoglobin assay kit (Nanjing Jiancheng Bioengineering Institute, Nanjing, China). Serum glucose, TG, TC, ALT (alanine aminotransferase), and AST (aspartate aminotransferase) were measured with commercially available assays purchased from Nanjing Jiancheng Bioengineering Institute. Serum insulin was measured with Ultra-Sensitive Mouse INS ELISA Kit (CUSABIO, Wuhan, China). All assays were completed according to the manufacturers' guidelines.

### 2.6. Liver Biochemistry Measurements

Hepatic lipids were extracted as described previously [33], when liver TG and liver NEFA content was determined using commercial kits (Applygen, Beijing, China; Nanjing Jiancheng Bioengineering Institute, Nanjing, China).

### 2.7. Liver Histopathology

Mice were sacrificed and perfused with saline via a portal vein to remove the blood. Then, liver tissues from all mice were fixed in 4% paraformaldehyde, dehydrated in graded ethanol, and embedded in paraffin. Five-micrometer sections were obtained using a microtome (Leica, Wetzlar, Germany) and stained with hematoxylin and eosin staining (ZSBG-BIO, Beijing, China) in accordance with the instructions. For oil red O staining, liver tissues that had been fixed in 4% paraformaldehyde were dehydrated in 20% and 30% sucrose solutions and embedded in optimal cutting temperature compound (SAKURA, Tokyo, Japan). Frozen sections were subjected to oil red O staining as per the manufacturer's instructions, and histopathological analyses were conducted by Service bio Laboratory (Wuhan, China).

### 2.8. RNA Extraction and Real-Time PCR

Total RNA was extracted from pulverized liver using TRIzol reagent (Invitrogen), followed by cDNA preparation from 1 ug of total RNA with a High-Capacity cDNA Reverse Transcription Kit (Takara, Japan). cDNA products were quantified by real-time PCR using Power SYBR Green PCR Master Mix (Applied Biosystems, Thermo, USA) on a StepOne Software v2.1 System (Applied Biosystems). We calculated mRNA using a 2^∧^deltaCT relative to the average of the *β*-actin expression. Detail of primers was listed in the Supplemental Table 2.

### 2.9. Western Blotting

Protein expression was measured in mice liver by Western blotting with antibodies obtained from Cell Signaling Technology (AMPK*α*, P-AMPK*α*^Thr172^) or from Santa Cruz Biotechnology (PPAR*α*, PPAR*γ*, *β*-actin) and Arigo (Anti-Rabbit IgG, Anti-Mouse IgG). Briefly, protein concentrations of total homogenate were determined by the Bradford method, and equivalent amounts of protein (20–40 *μ*g) were loaded into each lane of the SDS-PAGE gel (6–12%). After electrophoresis, proteins were transferred to 0.4 *μ*M PVDF membranes and stained with the Ponceau stain (0.1% wt/vol Ponceau Red in 5% acetic acid) to visualize protein loading. Membranes were washed with Tris-buffered saline containing 0.5% Tween 20 (1 *∗* TBST) and blocked for nonspecific binding room temperature (RT), 0.75–2 h, 5% (wt/vol) nonfat milk or BSA in TBST. Membranes were probed with primary antibodies for overnight at 4°C. After 3 times washing, the PVDF membranes were incubated with horse-radish peroxidase-conjugated secondary antibodies. Protein bands were quantified using FluorChem FC2 system (Alpha, Germany).

### 2.10. Quantification and Statistical Analysis

Results were analyzed using Student's* t*-test or ANOVA where appropriate, using GraphPad Prism software. A repeated-measures ANOVA was used for all body weight plots, GTT and ITT data. A Bonferroni's post hoc test was used to test for significant differences as determined by the ANOVA. *P* < 0.05 was considered significant. Data are presented as mean ± SEM.

## 3. Results

### 3.1. Ketogenic Diet and Ketogenic Diet Combined with Exercise Reduce Body Weights by Decreasing Food and Water Intake

**Figure 1:**
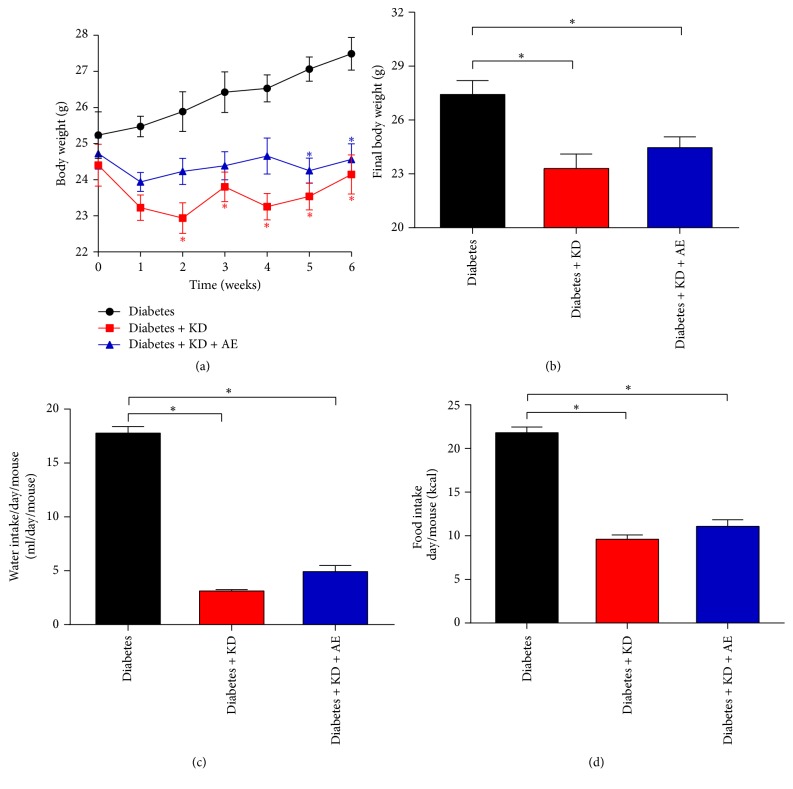
Ketogenic diet and ketogenic diet combined with exercise reduce body weights. (a) Changes in body weight during the 6-week treatment of ketogenic diet and aerobic exercise. Mice body weight of the diabetes + KD + AE group was significantly lower than that of the diabetes group, from the 5th week till the end. (b) Both ketogenic diet and a combination of aerobic exercise intervention could significantly decreased mice body weight. (c) Food intake and (d) water intake of diabetic mice were reduced after ketogenic diet or exercise. Data were presented as means ± SEM (*n* = 6 per group) and statistical analysis was performed with 1-way ANOVA. ^*∗*^*P* < 0.05 versus diabetic group.

The changes in body weight during the 6-week period of aerobic exercise and the final body weights are displayed in Figures 1(a) and 1(b). No significant differences were found in the baseline body weight of the three groups, while the body weight of the diabetes + KD group (from the 5th week till the end) and diabetes + KD + AE group (from the 2nd week till the end) was significantly lower than that of the diabetes group. Interestingly, mice treated with ketogenic diet with or without exercise both have lower food and water intake, compared with the diabetes group (Figures 1(c) and 1(d)), suggesting that the decrease in body weights by ketogenic diet intervention is due to reduced energy intake.

### 3.2. Ketogenic Diet and Ketogenic Diet Combined with Exercise Improve Insulin Sensitivity

**Figure 2:**
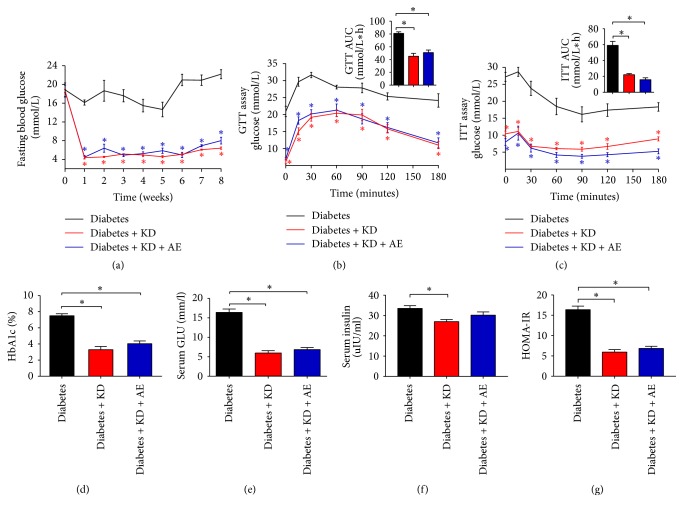
Ketogenic diet and ketogenic diet combined with exercise improve insulin sensitivity in diabetic mice. (a) Mice fasting blood glucose, (b) GTT, (C) ITT, (d) HbA1c levels, (e) serum glucose, (f) insulin levels, and (g) HOMA-IR indexes in the diabetes, diabetes + KD, and diabetes + KD + AE groups, after 6-week intervention. Data were presented as means ± SEM (*n* = 6 per group) and statistical analysis was performed with 1-way ANOVA. ^*∗*^*P* < 0.05 versus diabetic group.

To determine the effect of ketogenic diet and ketogenic diet combined with exercise on glycemic control in diabetic mice, we traced the dynamic metabolic parameters of each mouse. Compared with the diabetes group, fasting blood glucose levels in the diabetes + KD and diabetes + KD + AE groups were significantly decreased (Figure 2(a)). In addition, intraperitoneal glucose and insulin tolerance test were performed at the end of the 6-week experimental period. As expected, insulin sensitivity was improved significantly in the diabetes + KD and diabetes + KD + AE groups, compared with the diabetes group, as shown by GTT and ITT (Figures 2(b) and 2(c)). Furthermore, blood glycosylated hemoglobin (HbA1c) levels and serum glucose and insulin levels were also reduced significantly in mice treated with ketogenic diet and ketogenic diet (Figures 2(d)–2(f)). HOMA-IR is an indicator of insulin resistance. Thus, we calculate the HOMA-IR and found that the HOMA-IR indexes were significantly greater in diabetes than in diabetes + KD and diabetes + KD + AE groups (Figure 2(g)). Taken together, both ketogenic diet and ketogenic diet combined with exercise could improve insulin sensitivity and serum glucose parameters.

### 3.3. Ketogenic Diet and Ketogenic Diet Combined with Exercise Attenuate Hepatic Gluconeogenesis of Diabetic Mice

**Figure 3:**
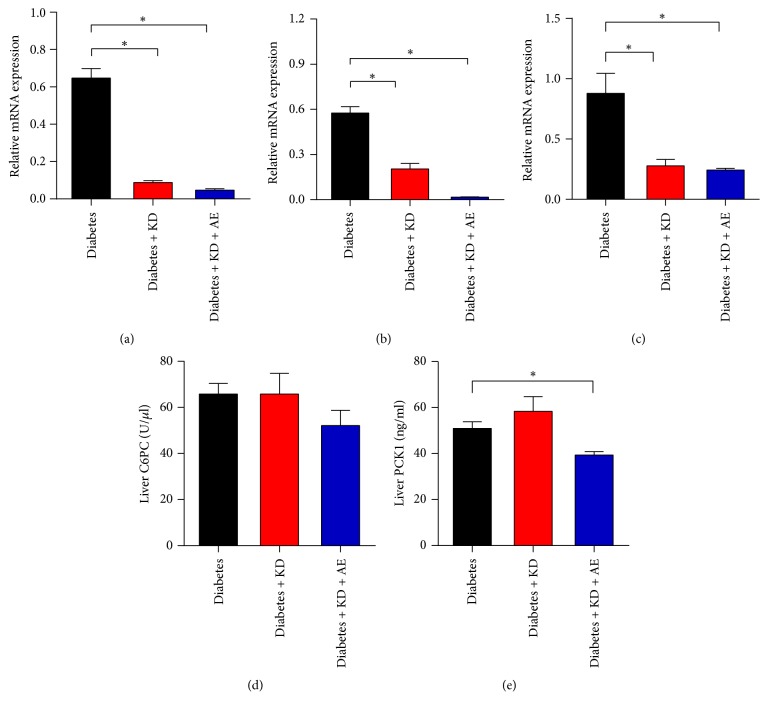
Ketogenic diet and aerobic exercise ameliorate hepatic gluconeogenesis of diabetic mice. (a) G6PC, (b) PCK1, and (c) FBPase mRNA expression levels in mice liver of the diabetes, diabetes + KD, and diabetes + KD + AE groups, after 6-week intervention. (d) G6PC and (e) PCK1 activity in mice liver of the diabetes, diabetes + KD, and diabetes + KD + AE groups, after 6-week intervention. Data were presented as means ± SEM (*n* = 6 per group). Statistical analysis was performed with 1-way ANOVA. ^*∗*^*P* < 0.05 versus diabetic group.

Our results showed that both ketogenic diet intervention and combination with aerobic exercise could improve glycemic control, while the mechanism by which factors regulate blood glucose homeostasis is not known. Liver gluconeogenesis is critical for maintaining stable blood glucose. Thus, we tested whether ketogenic diet with or without exercise may modulate gluconeogenic gene programs in livers of diabetic mice. G6PC, PCK1, and FBPase are critical rate-limiting enzyme of gluconeogenesis. We found that ketogenic diet and ketogenic diet combination with aerobic exercise significantly decreased the G6PC, PCK1, and FBPase levels in the liver (Figures 3(a)–3(c)). In addition, the activity of hepatic G6PC (Figure 3(d)) and PCK1 (Figure 3(e)) was slightly increased by ketogenic diet, while the activity of hepatic PCK1 was significantly suppressed by the combination intervention (Figure 3(e)). Thus, our data suggested that ketogenic diet and exercise could attenuate hepatic gluconeogenesis for improvements in glycemic control.

### 3.4. Aerobic Exercise Training Ameliorates Ketogenic Diet-Induced Metabolic Complications

**Figure 4:**
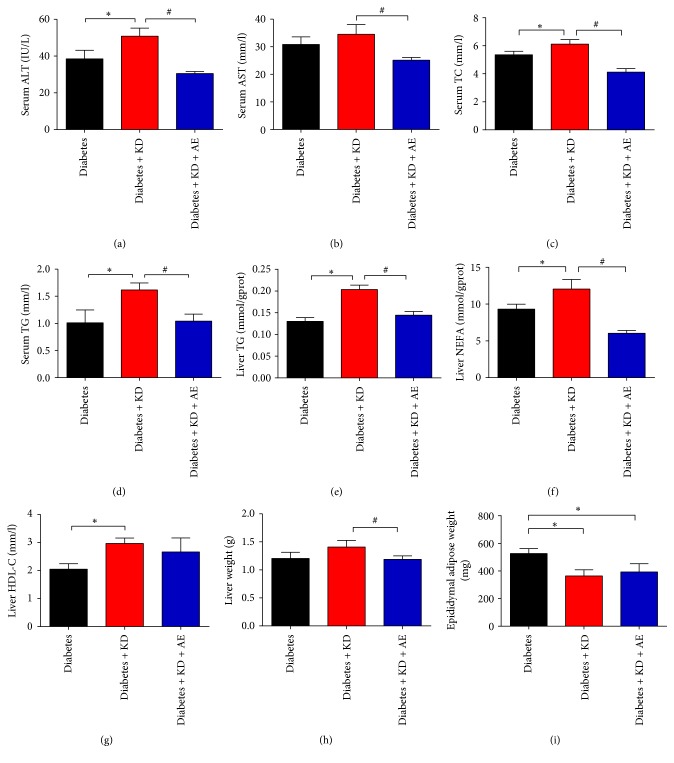
Aerobic exercise training improves ketogenic diet-induced metabolic complications.* Aim*. To evaluate the effects of ketogenic diet on metabolic parameters in diabetic mice. (a) ALT, (b) AST, (c) TC, and (d) TG levels in serum and (e) TG, (f) NEFA, and (g) HDL-C levels in mice liver of different groups were determined at the end of this experiment. (h) Liver and (i) epididymal adipose tissues are as a proportion of tissue weight per body. Data were presented as means ± SEM (*n* = 6 per group). Statistical analysis was performed with 1-way ANOVA. ^*∗*^*P* < 0.05 versus diabetic group and ^#^*P* < 0.05 versus diabetes + KD group.

Although ketogenic diet intervention is effective in reducing body weights and controlling glucose homeostasis, it showed lipid metabolic disorders after 6-week intervention. We found that ketogenic diet treated mice had significantly higher serum ALT, TC, and TG (Figures 4(a), 4(c), and 4(d)) compared with diabetes mice. However, the combination of ketogenic diets and exercise largely improved the impaired serum parameters caused by single treatment of ketogenic diets, such as serum ALT and TG (Figures 4(a) and 4(d)).

**Figure 5:**
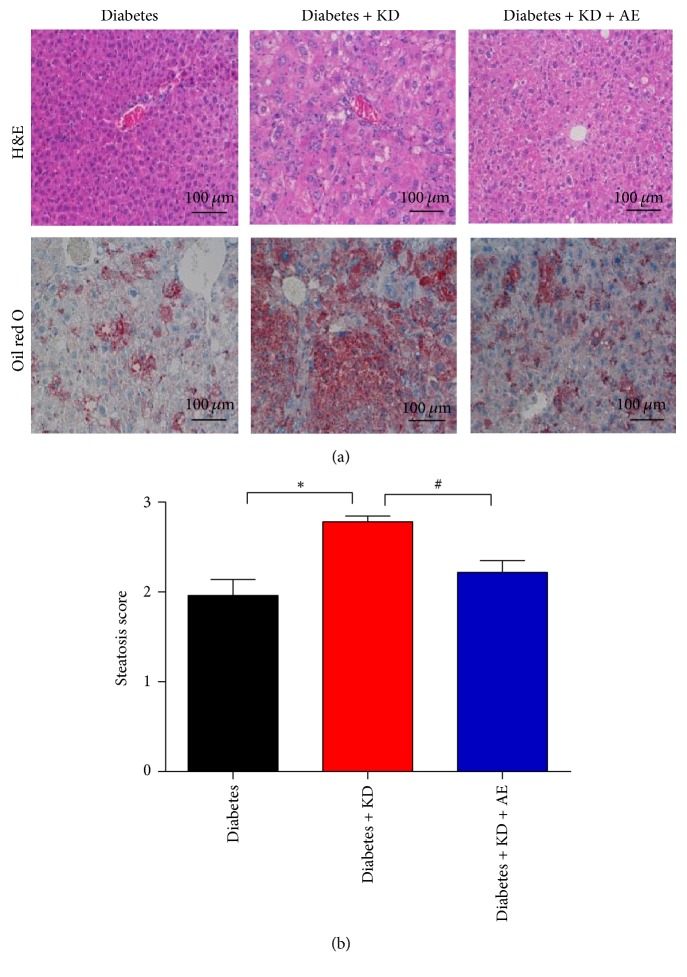
Aerobic exercise reduces ketogenic diet-induced hepatic steatosis in diabetic mice. (a) Representative images of hematoxylin and eosin and oil red staining of liver sections; (b) hepatic steatosis scores were evaluated based on oil red O staining. Original magnification, ×200. Scale bar, 100 *μ*m. Data were presented as means ± SEM (*n* = 5-6 per group) and statistical analysis was performed with 1-way ANOVA. ^*∗*^*P* < 0.05 versus diabetic group and ^#^*P* < 0.05 versus diabetes + KD group.

Ketogenic diets were reported to cause hepatic steatosis due to lipid dysregulation. We thus determined hepatic steatosis using biochemical analysis and morphological staining. The diabetes + KD group had more liver TG, NEFA, and HDL-c (Figures 4(e)–4(g)) as well as liver weight (Figure 4(h)) than the diabetes group, while the results of mice epididymal adipose tissue weight were different (Figure 4(i)). However, 6 weeks of aerobic exercise training largely attenuated hepatic steatosis in comparison with diabetes + KD as shown by liver biochemical parameters (Figure 5(a)), reduced fat droplets, and steatotic scores (Figures 5(a) and 5(b)). These results suggested that the combination of ketogenic diets and exercise largely reversed the adverse effects in lipid disorders and hepatic steatosis caused by single treatment of ketogenic diets.

### 3.5. Aerobic Exercise Modulates PPAR*α* and PPAR*γ* Gene and Protein Programs in the Liver

**Figure 6:**
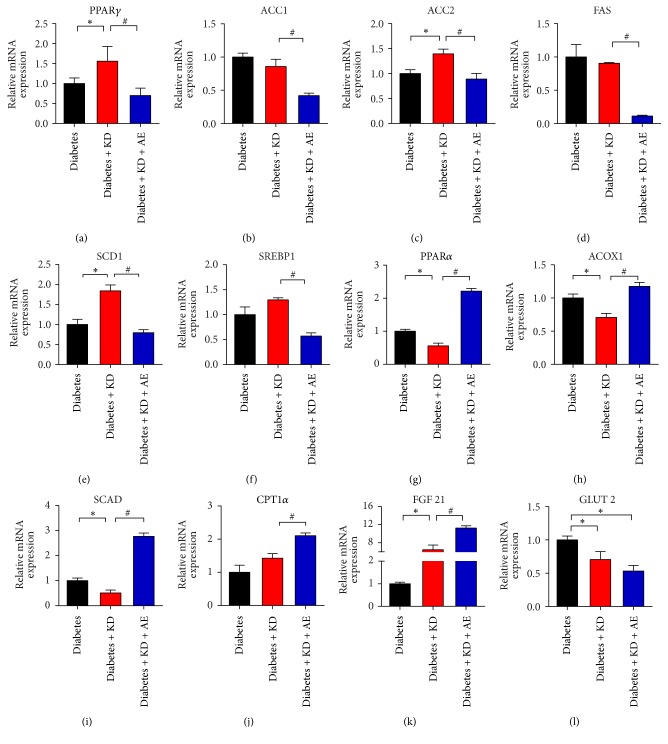
Aerobic exercise modulates PPAR*α* and PPAR*γ* gene programs in the mice liver. Gene expression of (a) PPAR*γ* and (b)–(f) its target genes in mice livers from diabetic group, diabetes + KD and diabetes + KD + AE groups, after 6-week diet and exercise intervention. Gene expression of (h) PPAR*α* and its target genes (g)–(j) in mice livers, from diabetic group, diabetes + KD and diabetes + KD + AE groups, after 6-week diet and exercise intervention. Gene expression of (k) FGF 21 and (l) GLUT2 is presented, after intervention. Results were examined by real-time PCR. Data were presented as means ± SEM (*n* = 6 per group) and statistical analysis was performed with 1-way ANOVA. ^*∗*^*P* < 0.05 versus diabetic group and ^#^*P* < 0.05 versus diabetes + KD group.

**Figure 7:**
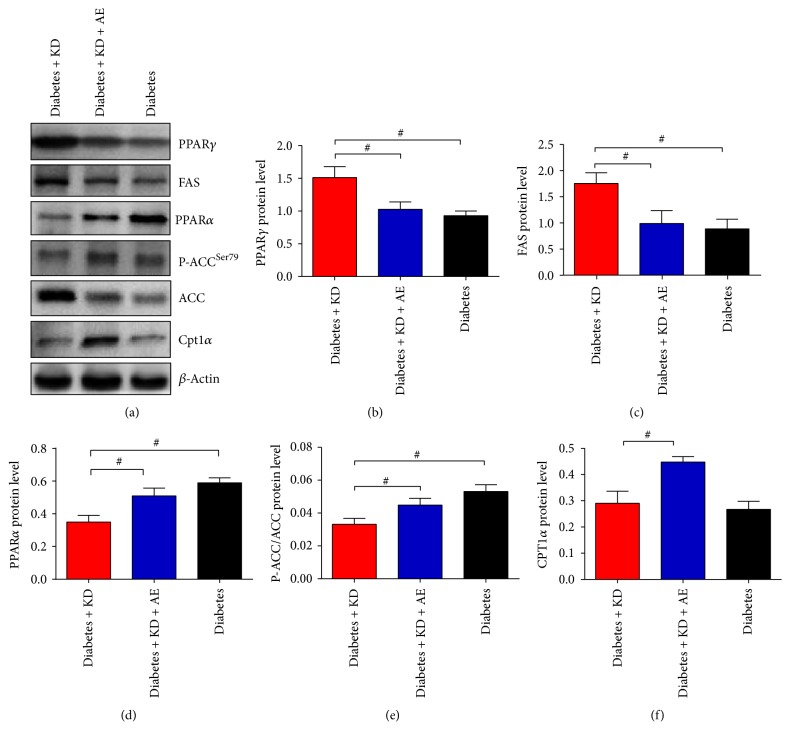
Aerobic exercise activates PPAR*α* and PPAR*γ* and target proteins in liver. Diabetic mice were treated with different diet with or without eight-week aerobic exercise (12 m/min 5 times/week). Liver tissue lysates were subjected to Western blot analyses with specific antibodies (a). The protein band intensity of (b) PPAR*γ*, (c) FAS, (d) PPAR*α*, (e) P-ACC/ACC, and (f) CPT1*α* was normalized to *β*-actin. Data were presented as means ± SEM (*n* = 6 per group) and statistical analysis was performed with 1-way ANOVA. ^*∗*^*P* < 0.05 versus diabetic group and ^#^*P* < 0.05 versus diabetes + KD group.

Next, we sought to determine the molecular mechanism to underneath the phenotype, since PPARs are critical for lipid metabolism in the liver. Specifically, PPAR***γ*** controls lipid storage and PPAR*α* regulates *β*-oxidation. Thus, at the molecular level, we examined the expressions of PPARs and their downstream metabolic genes in mouse livers by reverse transcription-polymerase chain reaction and found that PPAR***γ*** and its target genes, including ACC1, ACC2, FAS, SCD1, and SREBP1-C, were significantly higher in diabetes + KD mice while attenuated in the combination of KD and exercise (Figures 6(a)–6(f)). In addition, PPAR*α* and its *β*-oxidative target genes, such as Scad and Acox1, were significantly lower in diabetes + KD mice and the effects were lost by aerobic exercise in mice liver (Figures 6(h)–6(k)). Besides, we also observed that the liver Glut2 mRNA expression was reduced in KD and KD + AE groups, which suggested improved glycemic control in diabetic mice (Figure 6(l)). FGF21 promotes lipid catabolism, ketogenesis, and gluconeogenesis and improves insulin sensitivity and is an important target gene of PPAR*α* in the liver [34, 35]. We found that FGF21 levels were highly expressed in KD group and further elevated in KD + AE group, suggesting that elevated FGF21 levels may be responsible for the beneficial effects of AE in combination with KD (Figure 6(k)). Furthermore, the protein levels of key regulators of lipid and glucose metabolism were consistent with their mRNA levels (Figures 7(a)–7(f)).

AMPK is another key metabolic regulator in hepatocytes. We found that P-AMPK/AMPK levels exhibited similar change pattern as PPAR*α* did (Supplementary Figure 1), suggesting that exercise prevented ketogenic diets induced lipid disorders by decreasing lipid storage via PPAR*γ*, while increasing lipid *β*-oxidation via PPAR*α* and AMPK.

## 4. Discussion

Previous studies show that ketogenic diet has benefits on diabetes and might be used as a method of insulin resistance in diabetes. In this study, we demonstrate that the combination of ketogenic diet and aerobic exercise could improve glycemic control, while alleviating adverse effect of hepatic steatosis [36]. Furthermore, we found that after six weeks of intervention, mice treated with ketogenic diet and ketogenic diet combined with aerobic exercise both have lower body weights, HbAlc level, HOMA index, and higher insulin sensitivity [18, 21, 37, 38], compared with diabetes control group. However, our results also show that mice randomly assigned to the ketogenic diet had a variety of adverse effects including higher total cholesterol, triglycerides, and serum ALT and AST, compared to those of diabetes control group, where it can be largely reversed by aerobic exercise training [27, 29, 39]. Therefore, our data suggested that aerobic exercise combined with ketogenic diet may be a novel therapeutic approach for the treatment of nonalcoholic fatty liver disease and diabetes.

Almost a century has passed since the ketogenic diet was initially used in treatment of diseases, and advanced therapies based on ketogenic diets are now available for diabetes [40]. Previous studies have been concluded that restriction of dietary carbohydrates results in positive effects on metabolic and cardiovascular parameters [38, 41, 42]. The classic ketogenic diet is a high-fat, low-carbohydrate diet that induces ketone body production through fat metabolism with the goal of mimicking a fasting state in the body's tissues, shifting the predominant caloric source from carbohydrate to fat. This diet was used for a variety of health-related effects. Previous studies have reported that the ketogenic diet contributed to weight loss in diet-induced obese patients as well as improving glycemic control and metabolic parameters in diabetes. For instance, a low-carbohydrate ketogenic diet not only reduced blood glucose levels close to normal but also stabilized islet size and the number of *β* cells in diabetic rats [19, 21]. However, the potential side effects of a ketogenic diet are also cause for concern such as hyperlipidemia and hepatic steatosis [24, 25, 43]. In our study, we found that the hepatic steatosis was alleviated after combined ketogenic diet with aerobic exercise training, which could be a novel strategy for treating diabetes.

Nonalcoholic fatty liver disease (NAFLD) is considered the most common hepatic manifestation of metabolic syndrome affecting up to one-third of the adult population in affluent nations, and most obese individuals [44]. In China, approximately 20% of adults in the general population have NAFLD. Bland steatosis is considered to have a relatively benign prognosis as far as liver-related outcomes are concerned; those with steatohepatitis can progress to cirrhosis and its complications, including liver cancer [27]. Peroxisome proliferator-activated receptors (PPARs) were master lipid-activated transcription factors that play a key role in the regulation of lipid metabolism in liver, which belong to a class of nuclear receptors, because of their key role in the transcriptional regulation of mediators of glucose and lipid metabolism [45]. PPARs activate DNA transcription by binding to a relatively conserved DNA sequence in the vicinity of target genes. The group of PPARs consists of three distinct subtypes that differ in their expression profile across tissues and in their ligand specificity: PPAR*α*, PPAR*β*/*δ*, and PPAR*γ*.

In the liver, peroxisome proliferator-activated receptor *α* (PPAR*α*) functions at the master regulator of lipid metabolism, especially during fasting, by activating processes such as fatty acid oxidation and ketogenesis, fatty acid uptake, and triglyceride metabolism and storage [33]. The beneficial effects of PPAR*α* for NAFLD/NASH have been proven in several mouse models by the treatments of high-fat diet and trans-fat-rich diet. PPAR*α* null mice will develop a severe hepatic steatosis [46]. By encouraging flux through beta-oxidation and ketogenesis, PPAR*α* signaling limits the hepatic accumulation of lipids. This prevents hepatic oxidative stress that results from the generation of reactive oxygen species and lipid peroxidation products in response to excess hepatic lipid accumulation [47]. And, under periods of food deprivation, PPAR*α* promotes hepatic glucose and ketone production and prevents lipotoxicity. On the other hand, PPAR*γ* is a nutrition-induced factor in both adipogenesis and hepatic steatosis [48]. The expression of PPAR*γ* is induced in response to overnutrition in the liver. Furthermore, hepatocyte-specific PPAR*γ* expression is positively associated with fatty liver in mouse models [49]. For instance, overexpression of PPAR*γ* leads to hepatic steatosis and hepatocyte-specific knockout of PPAR*γ* reduces hepatic fat content in HFD-fed mice [50]. In this study, we found that aerobic exercise combined with ketogenic diet treatment could reduce the expressions of hepatic PPAR*γ* and its target genes including SREBP-1C, ACC1, SCD-1, and FAS, as well as increasing levels of PPAR*α* and its downstream target genes such as ACOX1, SCAD, CPT1-*α*, and FGF21, suggesting that PPARs might be at least partially responsible for the metabolic effects of the intervention.

De novo lipogenesis is a key mechanism for fat accumulation in the liver [51], which is often associated with the inhibition of AMP-activated protein kinase (AMPK) [52, 53]. AMPK is a key metabolic master switch in hepatocytes, activated by exercise [54]. Once activated, it will block anabolic pathways and promotes catabolic pathways and protects the cells from various stress stimuli, such as leading to increased fatty acid oxidation and suppression of fatty acid synthesis in hepatocytes [54]. AMPK activation further phosphorylated and inactivated ACC, which can modulate the proximal and rate-limiting step of lipogenesis [55]. In this study, we observed that aerobic exercise combined with ketogenic diet significantly increased AMPK phosphorylation, AMPK*α*1 and AMPK*α*2 protein abundance when compared with the ketogenic diet mice. Furthermore, aerobic exercise combined with ketogenic diet significantly increased hepatic PPAR*α* protein abundance and decreased PPAR*γ*, in comparison with the ketogenic diet group [56]. Previous studies thought that antihepatic steatosis effects of AMPK might be related to the regulation of proliferation key molecules in adipogenesis, such as PPARs. Taken together, the data obtained from our animal study indicates that aerobic exercise training combined with ketogenic diet ameliorates diabetes and hepatic and may involve activation of AMPK and PPARs pathways in liver [57–59].

Hepatic steatosis reflects hepatic oversupply of lipids, often induced through multiple mechanisms, including an increased flux of dietary and liberated visceral FA, increased hepatic FA synthesis, and reduced hepatic FA oxidation [60]. To date, there are no approved pharmacologic therapies for the treatment of NAFLD. Epidemiological studies suggest that exercise is a first-line therapy for patients with hepatic steatosis [29]. Impaired aerobic exercise capacity and skeletal muscle dysfunction appear to be associated with metabolic diseases, such as obesity, fatty liver, and diabetes. Also, numerous signaling pathways are involved in the initiation and progression of hepatic steatosis and related metabolic dysfunctions, and these pathways also interact with each other. In this research, we have focused on the role of AMPK/PPARs, while we cannot exclude the possibility that other metabolic factors may be involved in this process. Thus, further investigations using AMPK and PPARs knockout cells or mice models are useful to resolve these questions.

Traditional exercise guidelines have focused on low to moderate-intensity exercise because activities such as walking are easily achieved and relatively safe. However, such activities of daily living may not be able to provide an appropriate stimulus to increase cardiorespiratory fitness. High intensity interval training (HIIT), which involves brief bursts of vigorous exercise separated by periods of rest or recovery, has garnered attention in recent years in diabetes [61, 62] and NAFLD [29, 63, 64]. The present data show that the time-efficient HIIT may rapidly improve glucose control in diabetes and ameliorate hepatic steatosis. It needs further observation whether HIIT is more feasible than aerobic exercise for NAFLD patients with poor cardiorespiratory fitness or for those who cannot tolerate or participate in aerobic exercise.

Collectively, the data presented here suggest that aerobic exercise training combined with ketogenic diet may play a pathologically important role in regulating obesity, diabetes, and hepatic steatosis, which is largely dependent on the regulation of the AMPK/PPARs signaling.

## Supplementary Materials

Supplementary Materials Supplementary Figure 1: AMPK*α*1 and AMPK*α*2 levels in mouse livers of diabetic group, diabetes + KD group, and diabetes + KD + AE group. Supplementary Table 1: ingredient composition of diets used in the study. Supplementary Table 2: forward and reverse primers for real-time PCR used in the study.
