# Supplementary material for: Treatment of Diabetic Mice with a Combination of Ketogenic Diet and Aerobic Exercise via Modulations of PPARs Gene Programs
Source: PPAR Res. 2018 Mar 20;2018:4827643. doi: 10.1155/2018/4827643 (PMC5884211; doi:10.1155/2018/4827643)
Supplement: Supplementary Materials — Supplementary Figure 1: AMPKα1 and AMPKα2 levels in mouse livers of diabetic group, diabetes + KD group, and diabetes + KD + AE group. Supplementary Table 1: ingredient composition of diets used in the study. Supplementary Table 2: forward and reverse primers for real-time PCR used in the study. [file 4827643.f1.pdf]

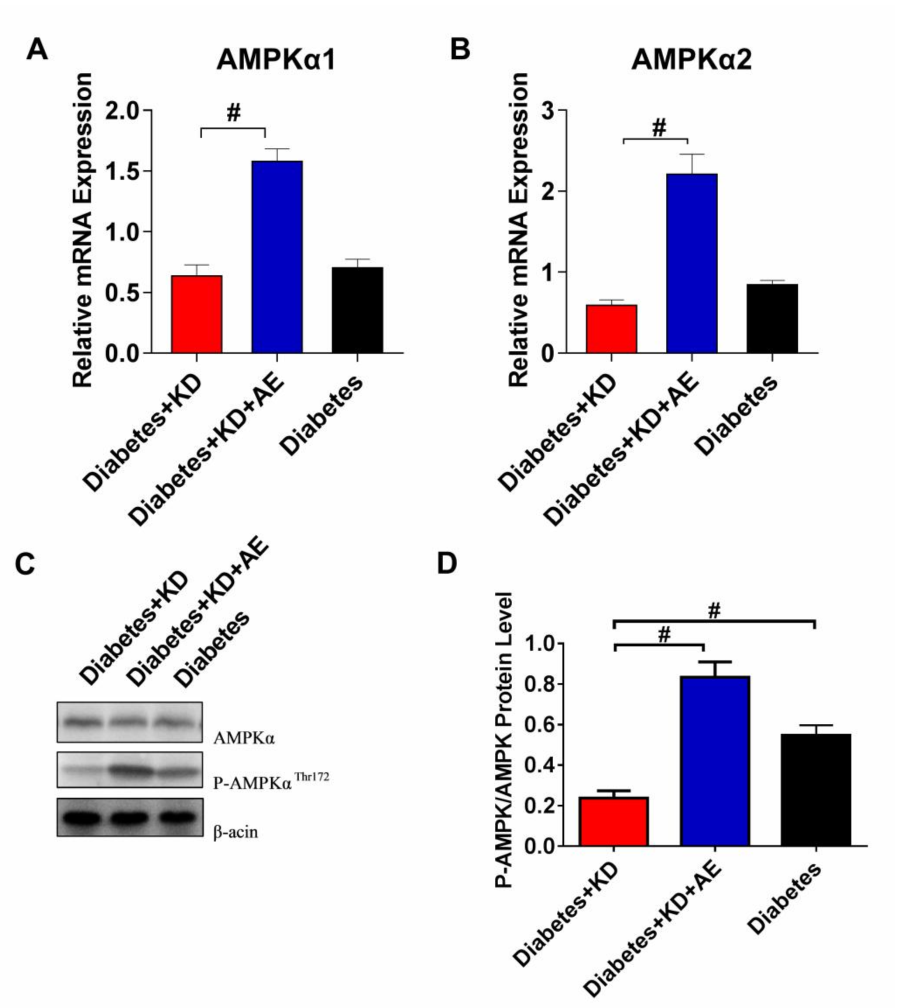

Supplementary Figure 1: AMPKα1 (A) & AMPKα2 (B) levels in mouse livers of diabetic group, diabetes+KD and diabetes+KD+AE group. AMPK protein expression and activity were shown below(C and D). Data were presented as means±SEM (n=6 per group) and statistical analysis was performed with 1-way ANOVA, #P<0.05 vs. Diabetes + KD group.

Supplemental Table 1：Ingredient composition of diets

g/100 g total diet	Chow diet	High-fat-diet	ketogenic Diet
Fish guano	4	2.18	-
Soybean meal	22	12.01	-
Corn	25	13.65	-
Wheat	34	18.56	-
Soybean	2	1.09	-
Bran	4	2.18	-
Yeast	2.5	0.82	-
Soybean oil	3	1.09	-
Lard	-	16.9	70
Casein	-	10.2	20
Sucrose	-	14	-
Maltodextrin	0.5	2.47	-
Choline bitartrate	0.2	0.11	-
Dietary fiber	-	-	5.5
Salt	0.4	0.4	0.38
Lysine	0.75	0.41	0.7
L-cystine	0.5	0.55	0.38
Mineral mix	0.15	0.26	0.14
Vitamin mix	0.2	0.2	0.19
Calcium hydrophosphate	1.6	1.57	1.5
Calcium carbonate	1.3	1.28	1.22

Nutrient composition: standard diet: protein (22.1%), carbohydrate (52%), fat (5.28%), fiber (4.12%); high-fat diet: protein (22.3%), carbohydrate (44.6%), fat (19.8%), fiber (2.1%); KD: protein (20%), carbohydrate (0%), fat (70%), fiber (5.5%).

Supplemental Table 2：Forward and reverse of gene

Product size/bp	Gene name	Primer Sequence（5'to3'）
155	AMPKa1-F	ACCTGACTCTTTCCTGGACG
	AMPKa1-R	AATGCCATTTTGCCTTCCGT
153	AMPKa2-F	CGCCTCTAGTCCTCCATCAG
	AMPKa2-R	TTGGGCTTCGTTGTGTTGAG
222	ACC1-F	TGAAGGCTGTGGTGATGGAT
	ACC1-R	CAGTAAGTGTAGGGTCCCGG
166	ACC2-F	TCTCTGAGGCCAACTGAACC
	ACC2-R	GGTCTCATCTGGCGTTCTCT
114	SCD1-F	AGAGTAGCTGAGCTTTGGGC
	SCD1-R	GCATCATTAACACCCCGATAGC
189	FASn-F	AGAGGCTTGTGCTGACTTCC
	FASn-R	CTCCACTCCCGAATGTGCTT
302	Acox1-F	GCTGAGGAACCTGTGTCTCT
	Acox1-R	TCAAAGGCATCCACCAAAGC
296	Scad-F	GGGCAAGAAGGAAGACAAGC
	Scad-R	CTCGGCATACTTCACAGCAC
157	PPARα-F	AGAGGGCTGAGCGTAGGTAA
	PPARα-R	ATTGGGCCGGTTAAGACCAG
162	PPARγ-F	CAGGTCAGAGTCGCCCCG
	PPARγ-R	CAGAGTGTGACTTCTCCTCAGC
83	CPT1a-F	ACGTTGGACGAATCGGAACA
	CPT1a-R	CCATGCAGCAGAGATTTGGC
173	G6PC-F	GGACCTCCTGTGGACTTTGG
	G6PC-R	TGCTGAGTTCTCCCTTGCAG
96	PCK1-F	CACTGACCCTCGAGATGTGG
	PCK1-R	CTGAGGCCAGTTTTGGGGAT
216	Glut2-F	CGTGTTCTTCCTCTTCGCTG
	Glut2-R	TGGTCGGTTCCTCGGTTTTA
315	FGF 21-F	CATACCCCATCCCTGACTCC
	FGF 21-R	TAGAGGCTTTGACACCCAGG
174	FBPase-F	CAGGGACGTGAAGATGAAGAAGAA
	FBPase-R	TTGTTGGCGGGGTATAAAAAGA
83	β-actin-F	GCAGGAGTACGATGAGTCCG
	β-actin-R	GGGTGTAAAACGCAGCTCAG
